# Difficulties in Management of Functional Movement Disorders: Three Illustrative Cases

**DOI:** 10.1002/mdc3.13264

**Published:** 2021-06-26

**Authors:** Martine Nadler, Isabel Cary, Christopher Symeon

**Affiliations:** ^1^ Department of Physiotherapy Wolfson Neurological Rehabilitation Centre, Queen Mary's Hospital (part of St George's Hospitals NHS Foundation Trust) Roehampton Lane London United Kingdom; ^2^ Department of Psychiatry Wolfson Neurological Rehabilitation Centre, Queen Mary's Hospital (part of St George's Hospitals NHS Foundation Trust) Roehampton Lane London United Kingdom

**Keywords:** FND, FEVD, novel treatment, rehabilitation

## Abstract

**Background:**

Some patients with FND and FEVD cannot re‐establish walking ability with standard treatment alone.

**Cases:**

Novel invasive treatment of FEVD trialed in three females, aged 19, 30 and 33 years with >18 month history of FND. None could walk and all were wheelchair‐dependent needing home carers. Standard treatment plus novel step‐wise escalation of invasive “intervention+” was individually tailored to correct FEVD; functional electrical stimulation, botulinum toxin injections, tibial nerve block, serial casting, and for Case 3, manipulation under anesthetic and surgical tendon lengthening. All regained walking ability and discontinued carers. Case 1 resumed dancing and Case 3 returned to employment. Improvements were largely maintained at 3 and 6 month follow‐up.

**Conclusions:**

As a last resort, invasive adjuncts may be considered in a very small proportion of FND patients who fail to regain walking ability with standard treatment alone and reach a “dead end” where no further progress is feasible.

Functional Neurological Disorder (FND) affects 16% of patients referred to neurology outpatient clinics.^1^ Symptoms can include sensory, motor and cognitive changes which occur in the absence of structural nervous system damage.

Sustained abnormal posture and activity can lead to joint deformity and soft tissue contracture. Fixed equinovarus dystonia (FEVD) is a musculoskeletal complication which presents a particular challenge to treat in patients with FND (Fig. 1). FEVD describes plantar flexion and inversion of the foot and ankle which is resistant to passive manipulation and in some/severe cases cannot be corrected. This deformity prevents the patient from being able to stand, weight‐bear or walk thus being wheelchair dependent. Correcting FEVD is essential before patients can embark on a program of gait re‐education.

**FIG. 1:**
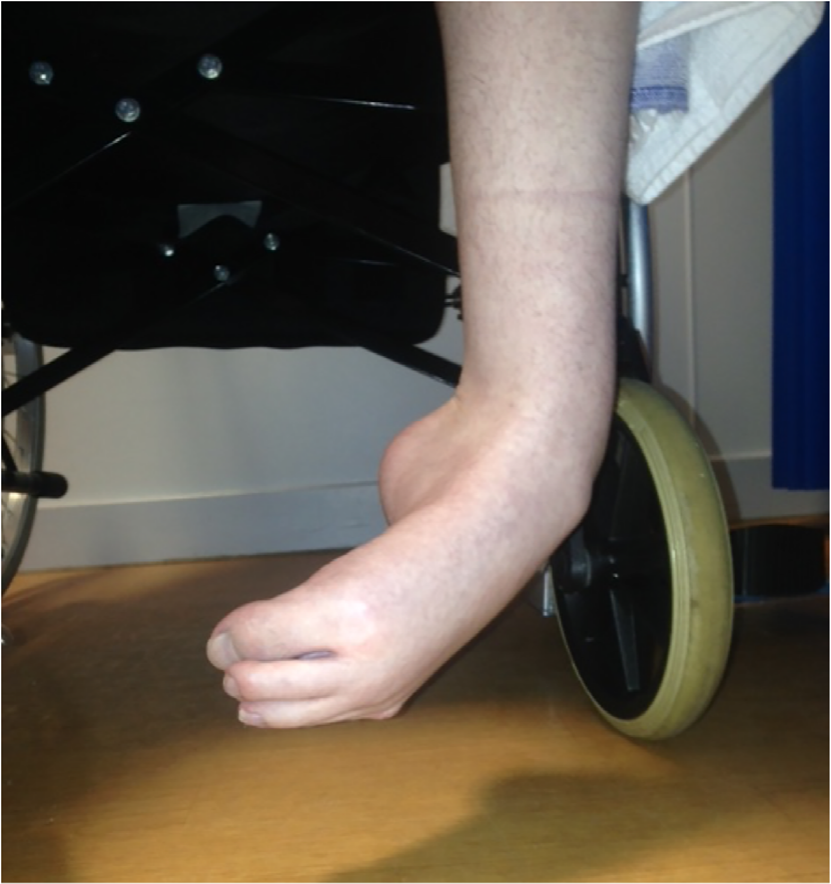
Case 3 showing FEVD.

Interventions targeted at the site of symptoms could be considered counter to recommended treatment models, where the goal is to retrain movement by diverting attention and focus from the affected area.^2^, ^3^ FEVD presents a conundrum as standard non‐invasive treatment may have limited effectiveness at correcting the sustained abnormal posture which may be associated with soft tissue and joint changes. We developed a novel approach which introduced progressively invasive treatments (Intervention+) delivered in a stepwise manner, supplementing standard treatment for selected patients with established FEVD. We present three case studies which may complement consensus opinion by demonstrating good physical outcomes using this approach.

## Case Series

### Setting

The Wolfson Neurological Rehabilitation Centre, Queen Mary's Hospital, London is a 28‐bedded unit providing specialist neurological in‐patient rehabilitation. Fifteen percent of patients admitted have FND with only three patients presenting with both, FND and FEVD during the past three years.

### Patient Cases 1–3 Overview

Three female patients, Cases 1, 2 and 3, aged 19, 30 and 33 were diagnosed with FND and FEVD by a neurologist and admitted for in‐patient rehabilitation between September 2017 and March 2019. All were wheelchair‐dependent for between 1.5 to 2.5 years and reliant on carers' support at home, a minimum of three times daily for personal and domestic activities. They were heavily reliant on analgesia medication including opioids to manage chronic pain symptoms. All gave informed consent to treatment, study publication and inclusion of anonymized photographs and video recordings.

### Intervention

Patients received standard treatment of patient‐led, goal oriented, multidisciplinary approach guided treatment. This is based on the consensus recommendation to address “illness beliefs, self‐directed attention and abnormal habitual movement patterns through a process of education, movement retraining and self‐management strategies within a positive and non‐judgmental context.”^3^

Cases 1, 2 and 3 were unable to progress to gait‐re‐education as part of their functional goals using standard treatment and as a last resort were screened to assess for suitability of “Intervention+” (Table 1). This is a step‐wise program with progressively more invasive treatment modalities and was discussed fully with the patient and multidisciplinary team (MDT).

**TABLE 1:** Standard treatment and intervention+ selected as appropriate for Cases 1, 2 & 3

**Standard treatment**	
**Standard Therapy co‐ordinated, consistent approach from multidisciplinary team**	**Patient‐identified functional, measurable targets/goals** **Education about FND**
Physiotherapy Occupational Therapy Speech & Language therapy Nursing Therapy Neuropsychiatry Neuropsychology	Enable self‐management for independence in personal and domestic activities of daily living Exercises to improve flexibility, mobility, specific muscle strengthening Pacing Stamina and fitness training Rhythm distraction techniques (Gait re‐education if effective weight‐bearing achieved) Assessment and treatment of co‐morbid psychiatric symptoms Psychological therapy (behavioral cognitive & empowerment approaches) Systemic interventions (family and environment) Program of rationalizing medication Pain management Support self‐management and relapse plan
**Intervention + (requires psychological and medical screening for suitability)**	
**Order of delivery of Intervention + to address gait re‐education**	
1. Functional electrical stimulation (FES)	Applied to tibialis anterior and peronei to address reversible FEVD position
2. Tibial nerve block (TNB) with Chirocaine/Levobupivacaine	Selective diagnostic nerve block to temporarily (up to 12 hours) suppress overactivity mainly in soleus, gastrocnemius, tibialis posterior, flexor digitorum longus and flexor hallucis longus to assess whether FEVD is reversible if FES inconclusive
3. Botulinum toxin A injections (BoNT A) using therapeutic doses to reduce overactivity (not placebo)	Gastrocnemius, soleus, tibialis posterior +/− flexor hallucis longus/brevis, flexor digitorum longus/brevis
4. Serial casting to ankle joint	to correct/maintain improved joint position
5. Manipulation under anesthesia (MUA)	Definitive test of correctability of FEVD prior to surgical intervention.
6. Surgical release of Achilles tendon, tibialis posterior, long toe flexors	To permanently correct joint position and maintained with further casting and orthotics

### Inclusion Criteria for Intervention+

1. Patient is fully engaged with therapy program
2. Patient accepts and understands the diagnosis of FND
3. Patient is highly motivated to walk and reduce wheelchair dependence
4. FEVD physical deformity is not reversible with standard treatment preventing weight‐bearing and gait re‐education
5. Patient is able to give informed consent for procedures
6. Patient has an on‐going rehabilitation goal of being able to walk which the clinical team believe may be achieved following improvement of FEVD

### Exclusion Criteria for Intervention+

1. Identification of significant secondary gain which has been identified as impacting on rehabilitation e.g. unresolved litigation, compensation claims or rehousing
2. Psychiatric illness which is impacting on the patient's health beliefs, including hypochondriacal disorders and delusional disorders
3. Acute mental health crisis
4. FEVD is resolving or improving with standard treatment

### Treatment Outcomes

All patients had failed to progress with standard treatment and therefore received different step‐wise levels of Intervention+. Case 1 required functional electrical stimulation (FES) to ankle muscles and botulinum toxin injections (BoNT) to correct FEVD. Case 2 demonstrated full ankle range following tibial nerve block indicating reversibility of FEVD which could not be achieved with FES and therefore serial casting was used to provide prolonged stretch to soft tissues (Supplementary Material S1). Case 3 trialed FES, BoNT, serial casting but FEVD was not reversible with these interventions. MUA did not change FEVD position due to severe, fixed, soft tissue contractures and therefore surgery was performed (Table 2 and Table S1).

**TABLE 2:** Stepwise intervention+ delivered to Cases 1, 2 & 3

**Case**	**Case 1**	**Case 2**	**Case 3**
	19 year old female dancer	33 year old mother	30 year old female PA
	5 year history symptoms	12 year history symptoms	4 year history symptoms
	2 year FND diagnosis	18 month FND diagnosis	3 year FND diagnosis
	19 week admission	12 week admission	24 week admission
**Admission: Mobility**	Wheelchair dependent 2 years	Wheelchair dependent 1.5 years	Wheelchair dependent 2.5 years (custom seating system)
**Discharge: mobility**	Independent walking no aids	Supervised walking with crutches	Independent walking with wheeled frame
**Pre‐admission care needs**	Carer QDS Hoist transfer Hospital bed Commode Downstairs living	Carer TDS Slide board transfer Downstairs living	24 hour carer (boyfriend) Turning aid transfer
**Discharge care needs**	Nil	Carer BD	Nil
**Six month follow up**	Returned to dancing No care needs No recurrence of FEVD	Became a volunteer Furniture walking Family support only No recurrence of FEVD	Returned to work Independent walking with crutches No care needs No recurrence of FEVD
**Intervention 1**	FES	FES	FES
**Intervention 2**	BoNT	TNB BoNT	TNB BoNT
**Intervention 3**	Not required	Ankle cast/splint serial casting	Ankle cast/splint serial casting
**Intervention 4**	Not required	Not required	Ankle manipulation under anesthetic
**Intervention 5**	Not required	Not required	Surgical release of Achilles tendon, tibialis posterior, long toe flexors

Details of stepwise intervention+ delivered to each Case and outcomes pre‐ and post‐admission and follow‐up.

FES, functional electrical stimulation to common peroneal nerve; BoNT, botulinum toxin to posterior tibial muscles; TNB, tibial nerve block; QDS, 4 times/day; TDS, 3 times/day; BD, 2 times.

**FIG. 2:**
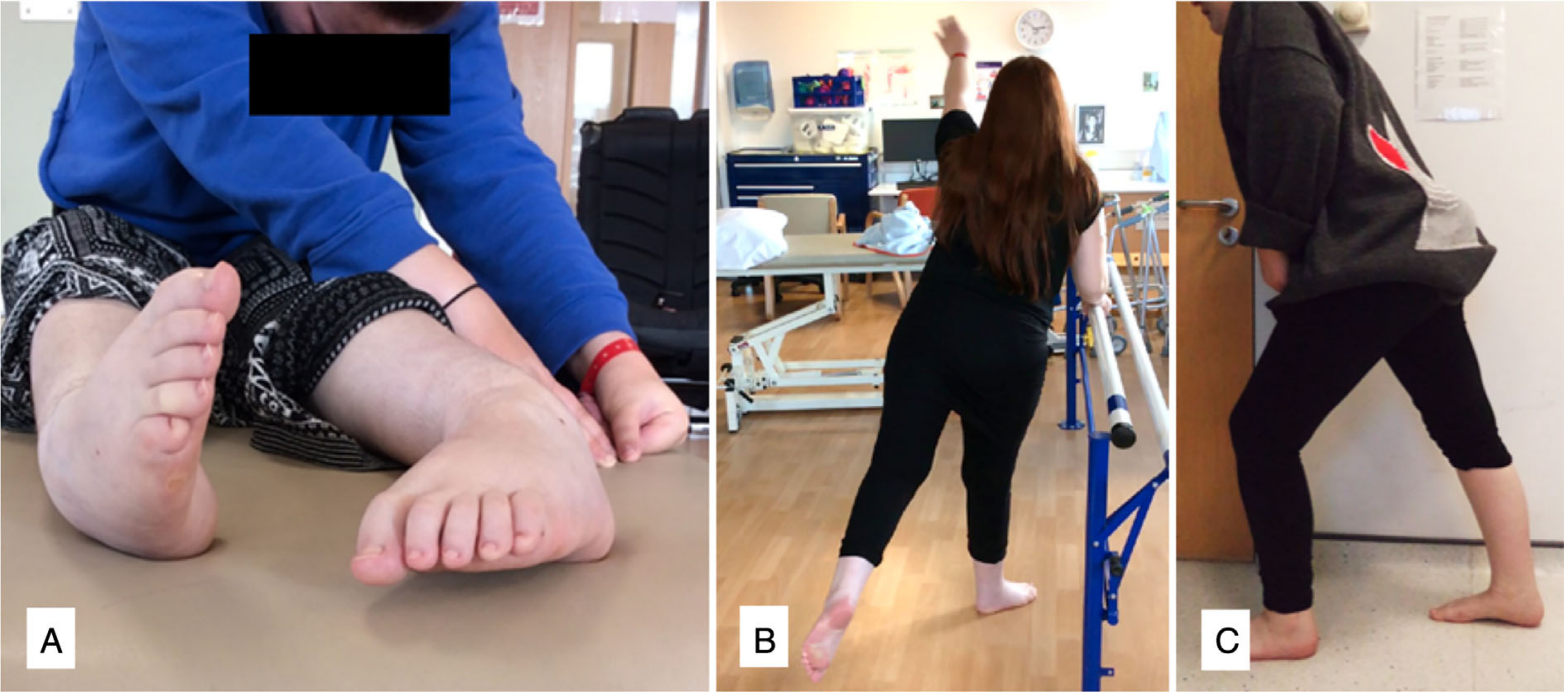
Case 1 (**A**) pre‐treatment and wheelchair dependent, (**B**) 3 weeks after Botulinum toxin (**C**) 4 months after Botulinum toxin.

**FIG. 3:**
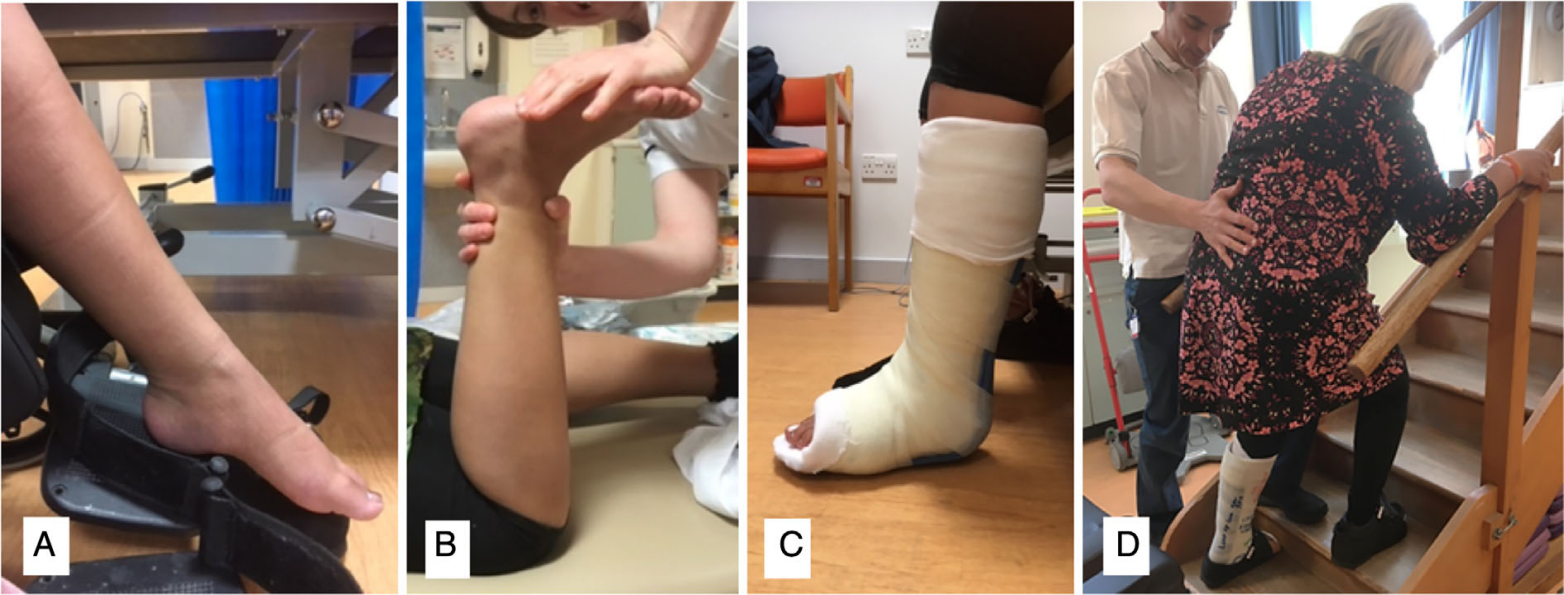
Case 2 (**A**) pre‐treatment and wheelchair dependent (**B**) position available immediately after tibial nerve block (**C**) first cast after tibial nerve block (**D**) second cast after tibial nerve block.

**FIG. 4:**
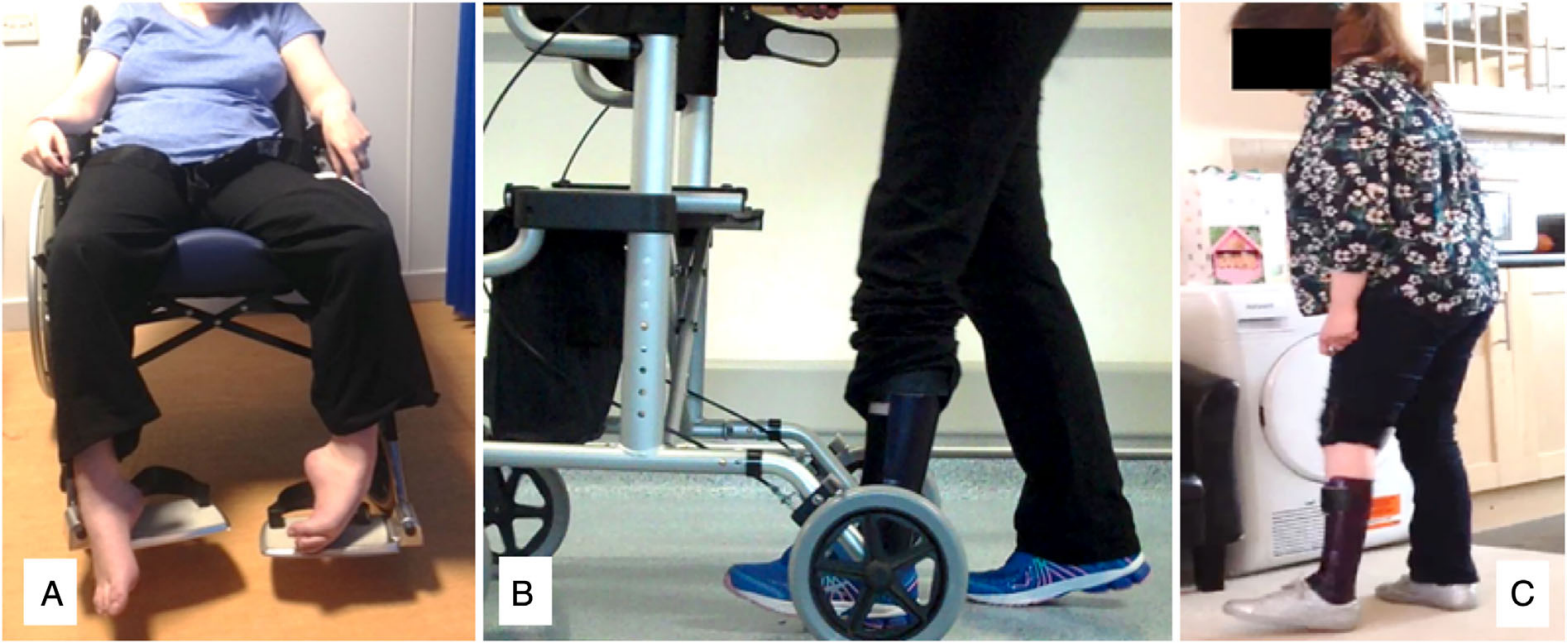
Case 3 (**A**) pre‐treatment and wheelchair dependent (**B**) 2 months after surgery (**C**) 5 months after surgery.

Wheelchair dependence was eliminated and all recovered the ability to walk (Video 1). Case 1 walked independently without aids and returned to dance classes (Fig. 2). Case 2 walked with an aid under supervision, re‐established active role as a mother/homemaker and explored volunteering (Fig. 3). Case 3 walked independently with an aid and returned to part‐time employment as a personal assistant (Fig. 4). Cases 1 and 3 no longer needed home carer visits and Case 2 had reduced care needs. All reported improved independence and quality of life (Table 2). In addition to physical gains, none required increased pain medication and Cases 1 and 3 successfully reduced opioid dependence

**Video 1:** Videos of Case 1 and Case 3 to show re‐established gait and weightbearing after intervention+.

### Six and Twelve Month Follow‐Up

None showed recurrence of FEVD at 6 months and all retained the ability to walk. At 12 months, Case 1 experienced some recurrence of mild, variable FEVD but this was correctable and she remained able to walk. Case 2 returned to baseline mobility levels but due to her improved psychological outlook she became an active volunteer in an international FND support group. Case 3 had a recurrence of flexion at the hallux which is a possible complication shown in stroke patients after equinovarus surgery.^4^ She continued to walk and is under regular orthopedic review.

## Discussion

The consensus for managing FEVD is overwhelmingly to use a non‐invasive approach for the majority of FND patients. Invasive interventions can be detrimental and cause worsening of symptoms.^3^, ^9^ We do not endorse invasive treatments as an obvious proactive step. However, the carefully selected cases presented here provide instructive exceptions to this rule as they failed to regain the ability to walk with non‐invasive approaches but responded to invasive intervention as a last resort. Treatment for FND varies considerably, depending on symptom presentation and the center providing treatment.^5^, ^6^ The standard treatment is MDT rehabilitation with an emphasis on cognitive behavioral therapy.^7^ Clinicians often de‐medicalize treatment where possible, encouraging patients to focus on more “normal” or “natural” movements and discourage using aids or adaptations as these can act as maintaining factors for abnormal posture and movement patterns.^8^ Unfortunately, access to specialist treatment for patients with FND often occurs late, and patients present with established abnormal movement patterns and may have musculoskeletal contractures, for example in FEVD. In this case series, standard treatment enabled all the patients to improve their general muscle strength, flexibility, stamina and increased independence in personal and domestic activities. However, all reached a “plateau” whereby the foot and ankle posture prevented them from weight‐bearing safely and effectively on that side. This precluded any possibility of their being able to walk again. This case series suggests that for a very small proportion of patients there may occasionally be a place for interventions of varying degrees of invasiveness as a last resort. These should always be discussed with the patient and carefully introduced in a step‐wise progression to address the FEVD effectively enabling them to overcome the plateau. It is extremely rare for surgery to be indicated as in Case 3. Screening was performed by a consultant neuropsychiatrist to ensure that these patients selected for more invasive procedures were not suffering from a significant comorbid psychiatric disorder which could affect or be affected by escalated medical intervention. All patients were extremely motivated to walk again and were central to the decision‐making process about any treatment options offered to enable them to work towards this goal. Suitability screening was carried out to identify potential to change the physical maladaptation. Reversibility of FEVD was key in choosing progressively more invasive interventions with surgery deemed appropriate only for Case 3. This was discussed with her as manipulation under anesthetic failed to change her FEVD and she was highly motivated to walk. Since admission, she improved from being semi‐reclined in a wheelchair with a custom matrix postural seating system to being able to hop with crutches but remained unable to weight‐bear due to FEVD on the affected leg.

Some patients with FEVD have a pre‐disposing peripheral injury^9^ which may be an indicator of poor outcome; our small cohort did not present with this. They reported pain but none presented with Chronic Regional Pain Syndrome (CRPS) features of allodynia, sudomotor, temperature or color changes. Allodynia may be an important factor for tolerability of more invasive interventions such as rigid casting, FES, or BoNT injections. In general, caution should be exercised when considering invasive treatment adjuncts as FND symptoms may be exacerbated.^9^ Schrag and colleagues reported deterioration of dystonia in a proportion of these patients following casting.^9^ To minimize this risk we ensured that patients understood the rationale for casting and were keen to proceed, they were given adequate pain relief for the duration of casting and monitored throughout with the ability to remove cast immediately should they become unable to tolerate it.

### Additional Considerations

Patients with FND and FEVD remain vulnerable to deterioration. Therefore, it is vital that they have access to ongoing follow‐up if rehabilitation gains are to be maintained in this complex cohort of patients. Surgical intervention is rare for FND patients with FEVD. This is because there have been case reports of patients undergoing surgery for severe pain resulting from fixed dystonia which has caused these dystonic postures to present elsewhere in the body.^10^ In Case 3, the possibility of this occurring after surgical release of soft‐tissue contracture was explained to her and she gave informed consent to proceed. Carefully timed intensive rehabilitation post‐surgery is of paramount importance together with well‐planned pain relief pre‐ and post‐operatively. The intervention+ approach should be considered only after progress using standard treatment has plateaued. Careful assessment and patient selection is essential. Inclusion and exclusion criteria given above may provide a framework to guide clinical decision making for this novel step‐wise intervention+ approach. Additionally, absence of allodynia or pre‐existing peripheral injury may be important indicators for patients most likely to tolerate intervention+ and re‐establish walking ability. Service considerations include the ability to provide specialist in‐patient care and close monitoring and support by an MDT experienced with treating patients with FND for as long as is required. If any of the above considerations are not addressed then invasive interventions may be unsuccessful or even detrimental.

### Conclusions

As a last resort, invasive adjuncts may be appropriate in a very small proportion of FND patients with FEVD who fail to progress with standard treatment alone and reach a “dead end” where no further progress is feasible.

It is vital that careful psychological and medical screening is carried out before considering the intervention+ pathway described for FEVD in FND patients. If appropriate, this must only be provided in a specialist, in‐patient setting with close monitoring by the MDT. As a last resort for these three cases who had stopped responding to standard treatment, the invasive intervention enabled them to walk again.

## Author Roles

(1) Research project: A. Conception, B. Organization, C. Execution; (2) Statistical Analysis: A. Design, B. Execution, C. Review and Critique; (3) Manuscript: A. Writing of the first draft, B. Review and Critique; (4) Manuscript Revision: A. Rewriting, B. Review and Critique.

M.N.: 1A, 1B, 1C, 3A, 4A

I.C.: 1A, 1B, 1C, 3B, 4B

C.S.: 1A, 1B, 1C, 3B, 4B

## Disclosures

### Ethical Compliance Statement

The authors confirm that the approval of an institutional review board was not required for this work. The authors confirm that all three patients gave informed consent for the use of anonymized photographs and video recordings for this work. We, the authors, confirm that we have read the Journal's position on issues involved in ethical publication and affirm that this work is consistent with those guidelines.

### Funding Sources and Conflicts of Interest

The authors did not receive any funding for this study. The authors have no conflicts of interest to declare.

### Financial Disclosures for the Previous 12 Months

The authors have no conflicts of interest to declare for the previous 12 months.

## Supporting information

**Supplementary Material S1.** Serial Casting Case 2 and Case 3.

**Table S1.** Case 3 Gantt Chart Timeline Intervention+.
