# Supplementary material for: Difficulties in Management of Functional Movement Disorders: Three Illustrative Cases
Source: Mov Disord Clin Pract. 2021 Jun 26;8(6):932–9. doi: 10.1002/mdc3.13264 (PMC8354088; doi:10.1002/mdc3.13264)

# Casting: General Guidelines

For full splinting guidelines and precautions please refer to:

https://www.acpin.net/pdfs/Splinting_Guidelines.pdf
https://www.acpin.net/pdfs/Neurosplinting_Quick_Reference_Guide.pdf

**Brief summary:**

**Indications for casting to correct FEVD**

Provide sustained stretch of ankle soft tissues to increase range of ankle dorsiflexion and enable foot placement on floor for walking.

**Additional Precautions**

Cast application should be performed by a therapist with specialist skills when patients are undergoing in-patient rehabilitation to enable close monitoring.

Apply cast at 5-10 degrees less than maximal, available range. Do not cast at maximal end of range of dorsiflexion as this is extremely uncomfortable and cannot be tolerated by the patient.

Ensure toes are visible to monitor circulation.

Ensure ward nursing staff understand how to monitor cast and remove if required when therapy staff are not available.

Splinting scissors should be made available at all times to enable cast to be removed without delay if patient becomes distressed and to avoid iatrogenic harm.

**Case 2 –Following single post-tibial nerve block (TNB); two non-removeable, serial, walking casts applied consecutively each for two weeks duration.**

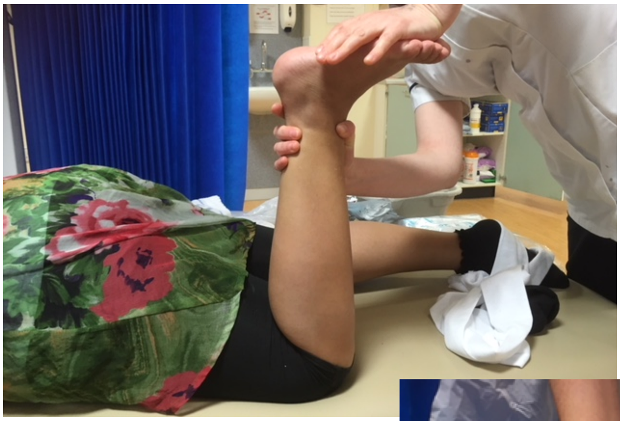

Plantigrade position achieved immediately after TNB.

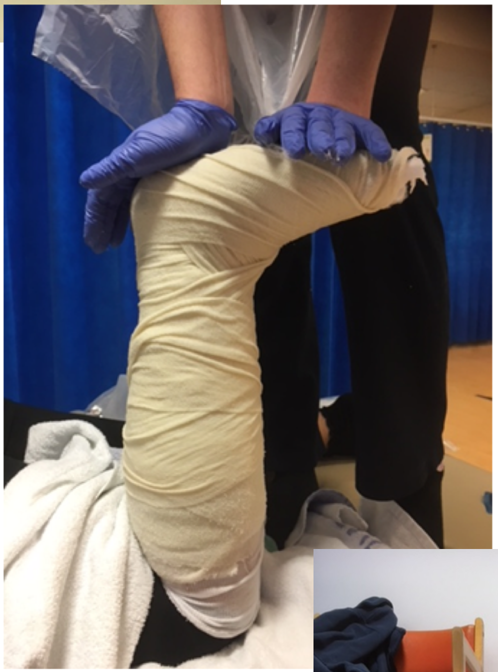

*Softban Pure*® cotton bandage applied under 3M *Softcast*® and 3M *Scotchcast*® to reduce risk of pressure areas.

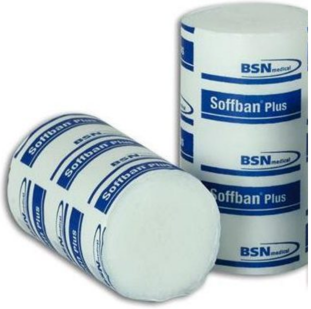


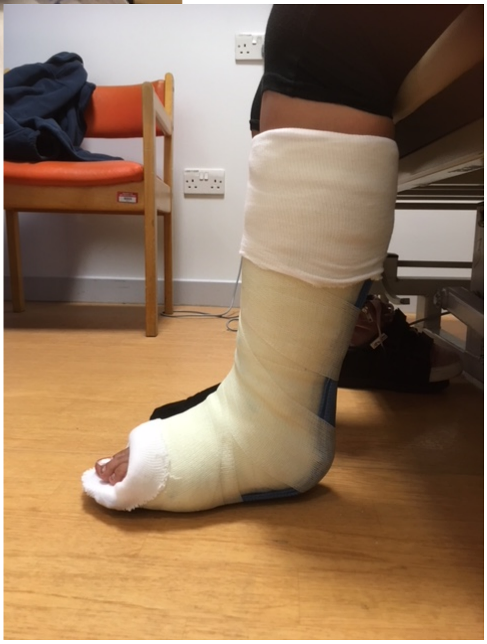

Non-removable serial casts applied at 10 degrees plantar flexion for sustained stretch. This was 5 degrees from maximal available range of ankle dorsiflexion.

**Case 3 – Following one cycle of Botulinum Toxin injections; Four serial casts applied consecutively with each cast non-removeable for 24-48 hours then removeable for 7 days.**

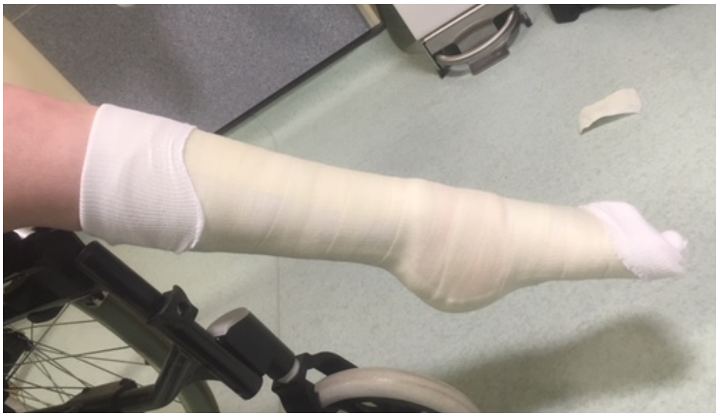

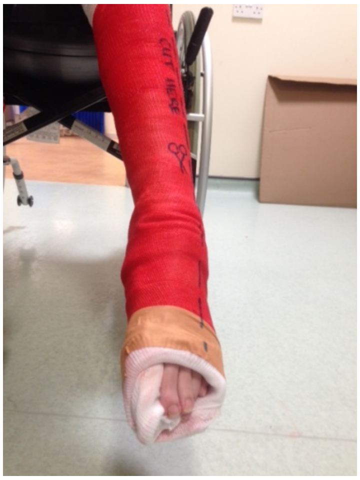

Non-removeable serial cast with *Softban Pure®* cotton bandage applied under 3M *Softcast®* and 3M *Scotchcast®* 5 degrees from maximum available range of dorsiflexion.

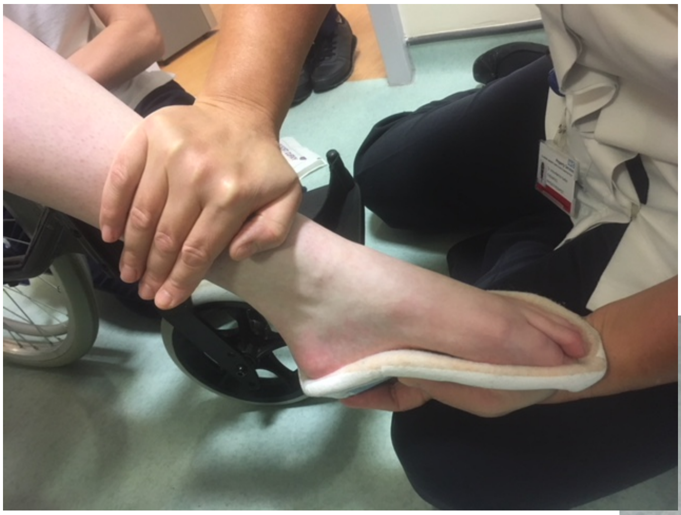

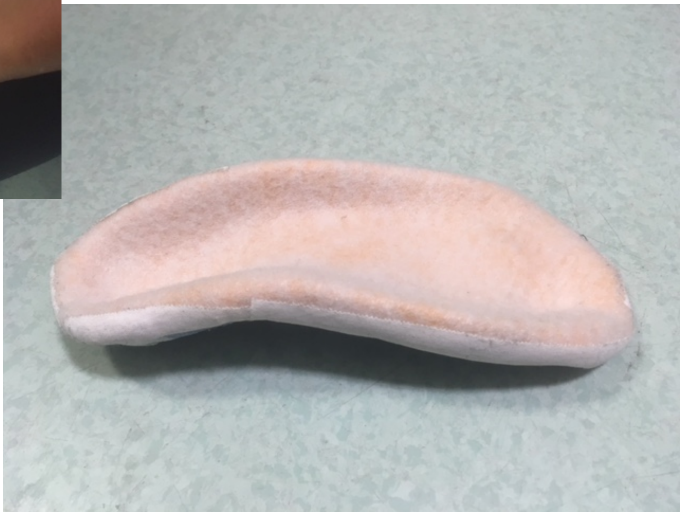

Removable padded moulded insole was bandaged on to maintain stretch on toes for periods when resting out of removable cast.

**Case 3 –Comparison of consecutive casts to show minimal improvement in range of ankle dorsiflexion (A) which was accompanied by worsening range of ankle inversion (B)**

Dotted line indicates position to cut 3M *Softcast®* to convert non-removeable cast into a removeable cast.

**A**

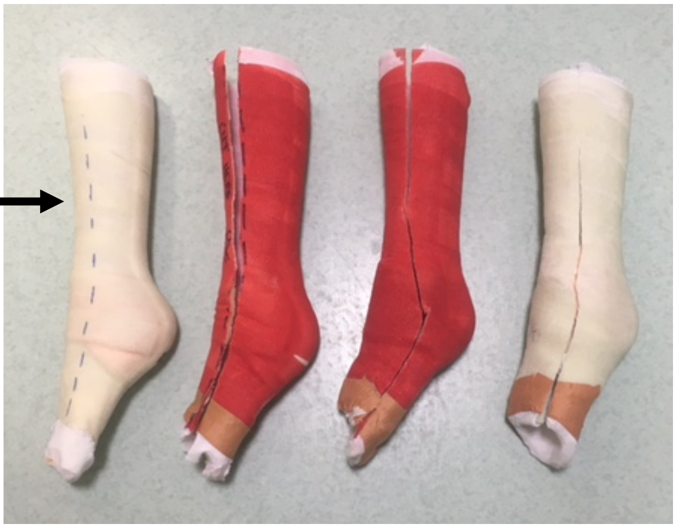

1 2 3 4

**B**

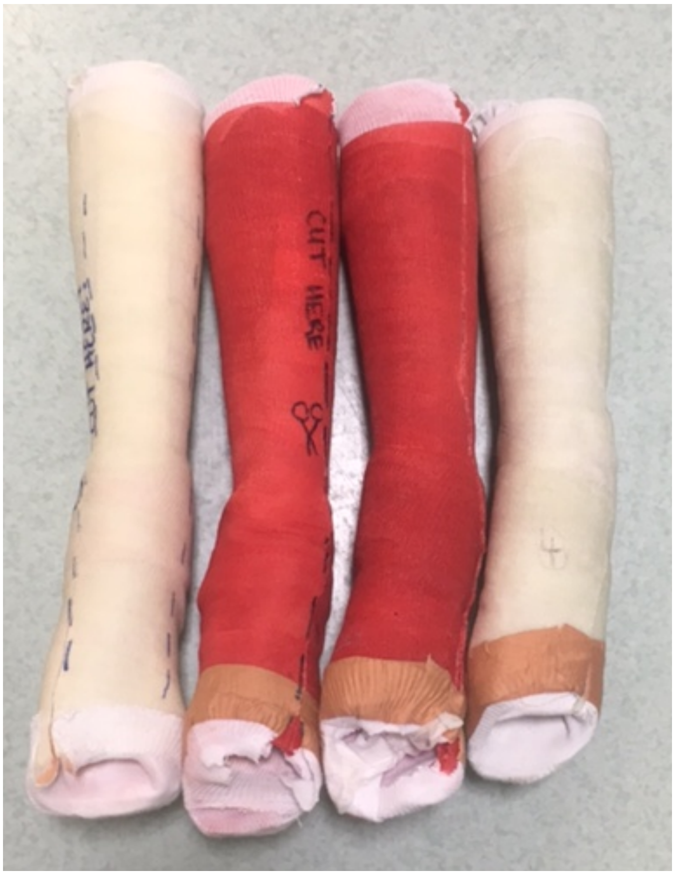

Supplement: Supplementary file 1 — Supplementary Material S1. Serial Casting Case 2 and Case 3. [file MDC3-8-932-s002.pdf]
