# Supplementary figures and images for: Difficulties in Management of Functional Movement Disorders: Three Illustrative Cases
Source: Mov Disord Clin Pract. 2021 Jun 26;8(6):932–9. doi: 10.1002/mdc3.13264 (PMC8354088; doi:10.1002/mdc3.13264)

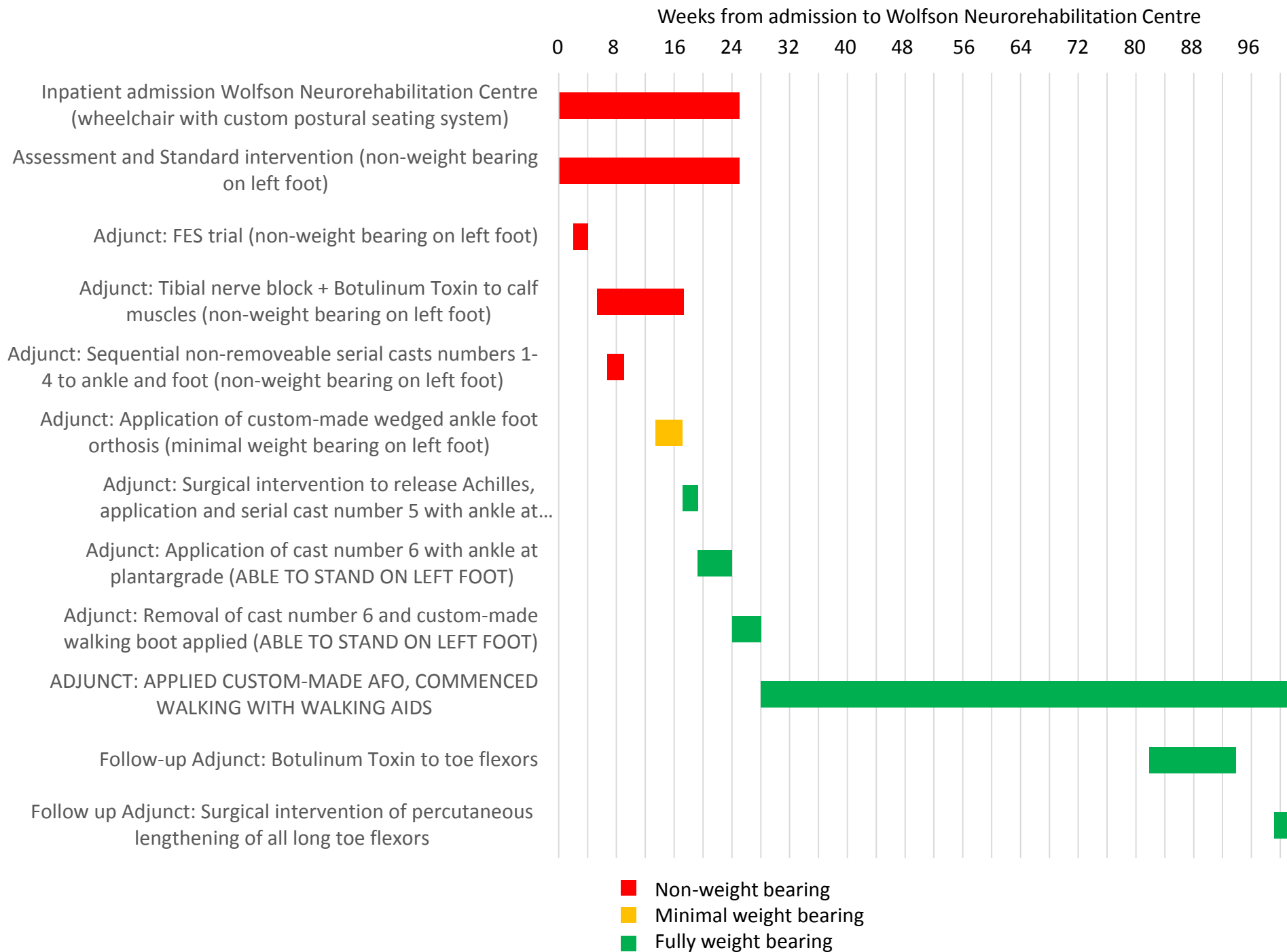

Supplement: Supplementary file 2 — Table S1. Case 3 Gantt Chart Timeline Intervention+. [file MDC3-8-932-s001.pdf]
